# Antidepressant and Anti-Neuroinflammatory Effects of Bangpungtongsung-San

**DOI:** 10.3389/fphar.2020.00958

**Published:** 2020-07-10

**Authors:** Bo-Kyung Park, No Soo Kim, Yu Ri Kim, Changsop Yang, In Chul Jung, Ik-Soon Jang, Chang-Seob Seo, Jeong June Choi, Mi Young Lee

**Affiliations:** ^1^Clinical Medicine Division, Korea Institute of Oriental Medicine, Daejeon, South Korea; ^2^Department of Oriental Neuropsychiatry, College of Korean Medicine, Daejeon University, Daejeon, South Korea; ^3^Division of Bioconvergence Analysis, Korea Basic Science Institute, Daejeon, South Korea; ^4^K-herb Research Center, Korea Institute of Oriental Medicine, Daejeon, South Korea; ^5^Laboratory of Molecular Medicine, College of Korean Medicine, Daejeon University, Daejeon, South Korea

**Keywords:** reserpine, depression, Bangpungtongsung-san, antidepressant, anti-neuroinflammation, neuroprotection, BV2, microglia

## Abstract

Bangpungtongsung-san (BTS) is a traditional Korean medicine consisting of 18 herbs, some which have antidepressant effects. Here, we used an animal model of reserpine-induced depression and lipopolysaccharide (LPS)-stimulated BV2 microglia to assess the antidepressant and anti-neuroinflammatory effects of BTS. Aside from a control group, C57BL/6 mice were administered reserpine (0.5 mg/kg) daily for 10 days *via* intraperitoneal injection. BTS (100, 300, or 500 mg/kg), vehicle (PBS), or fluoxetine (FXT, 20 mg/kg) was administered orally 1 h before reserpine treatment. Following treatment, a forced swimming test (FST), tail suspension test (TST), and open field test (OFT) were performed, and immobility time and total travel distance were measured. Administration of BTS not only reduced immobility time in the FST and TST but also significantly increased the total travel distance in the OFT. Furthermore, reserpine-treated mice showed significantly elevated serum levels of corticosterone, a stress hormone; however, treatment with BTS significantly reduced corticosterone levels, similar to FXT treatment. Serotonin in reserpine-treated mice was significantly reduced compared to that in control mice, while BTS mice exhibited increased serotonin levels. BTS mice showed increased expression of brain-derived neurotrophic factor (BDNF) and a higher ratio of phosphorylated cAMP response element-binding protein (p-CREB) to CREB (p-CREB/CREB) in the hippocampus. Additionally, reserpine-treated mice exhibited significantly elevated mRNA levels of pro-inflammatory cytokines, but BTS mice showed reduced mRNA levels of interleukin (*IL)-1β, IL-6*, and tumor necrosis factor (*TNF)-α* in the hippocampus. To further demonstrate the anti-neuroinflammatory effects of BTS *in vitro*, we examined its anti-neuroinflammatory and neuroprotective effects in lipopolysaccharide (LPS)-stimulated BV2 microglia. BTS significantly reduced the levels of NO, inducible nitric oxide synthase (iNOS), cyclooxygenase (COX)-2, TNF-α, IL-1β, and IL-6 in a dose-dependent manner *via* a decrease in the expression of nuclear factor (NF)-κB p65. Furthermore, the neuroprotective factor heme oxygenase-1 (HO-1) was upregulated *via* the nuclear factor-E2-related factor 2 (NRF2)/CREB pathway. Taken together, our data suggest that BTS has considerable potential as an anti-neuroinflammation and antidepressant agent, as it has clear effects on depressive behaviors and associated factors caused by reserpine-induced depression

## Introduction

Depression is an emotional disorder associated with various symptoms, such as sleep disorders, eating disorders, and anxiety (Organization, 2009). Major depressive disorder (MDD) is defined as the persistence of a depressed mood for at least 2 weeks or the loss of pleasure or interest in activities that are usually found enjoyable (Kessler et al., 2003). MDD has a lifetime prevalence of around 15%–25% worldwide and is among the highest ranked diseases according to the World Health Organization (WHO) disease burden ranking (Greenberg et al., 2003; Simon, 2003).

The causes of depression include environmental as well as genetic factors, but environmental aspects related to urbanization and industrialization are largely responsible for the observed increase in prevalence (Hidaka, 2012). The classic model for the pathogenesis of depression is based on the monoamine hypothesis (Tiemeier, 2003). In the 1960s, a neurochemical model of depression was proposed, based on a report that monoamine depletion caused adverse effects leading to depression in patients using reserpine for treatment of hypertension (Schildkraut, 1965). This demonstrated that monoaminergic dysfunction in the central nervous system is associated with depression and led to the development and study of associated tricyclic antidepressants (TCAs), selective serotonin reuptake inhibitors (SSRIs), noradrenaline reuptake inhibitors (NRIs), serotonin and noradrenaline reuptake inhibitors (SNRIs), monoamine oxidase inhibitors (MAOIs), and other antidepressants (Torres et al., 2003). However, these antidepressants are known to cause side effects such as dizziness, sedation, anticholinergic side effects, weight gain, sexual dysfunction, neurological side effects, cardiovascular effects, insomnia, and anxiety (Sarko, 2000). To overcome these limitations, recently, modulators of neuroinflammation, oxidative stress, the hypothalamic pituitary adrenal axis, glutamate, and opioids, as well as anticholinergic drugs and some neuropeptides such as substance P, neuropeptide Y, and galanin, have been developed, and various attempts have been made to treat depression without side effects (Papakostas and Ionescu, 2015; Rosenblat et al., 2015).

Bangpungtongsung-san (BTS) is a traditional Korean medicine described in the Donguibogam, a well-documented textbook of traditional Korean medicine (Jun, 1980; Song et al., 2016). It was discovered while searching for a prescription for depression and comorbid diseases and acts as a 5-hydroxytryptamine (5-HT)_2C_ antagonist in 5-HT_2C_ receptor binding assays of our screening test. BTS has been approved by the Korean Ministry of Food and Drug Safety (MFDS) and has been widely used in Japan and Korea for the treatment of diseases such as hypertension, obesity, allergic rhinitis, and atherosclerosis (Lee M.Y. et al., 2012). In addition, some studies have reported that BTS treatments improve immune function. BTS downregulated the MAPK (ERK, JNK, and p38) and NF-κB signaling pathways in macrophages stimulated by LPS, thereby reducing inflammation-inducing mediators such as NO, PGE2, TNF-α, and IL-6. Furthermore, the anti-inflammatory effect of BTS was demonstrated to reduce edema and sensitization *in vivo* (Lee C.W. et al., 2012). BTS is composed of the herbs *Angelica gigas, Paeonia lactiflora, Cnidium officinale, Gardenia jasminoides, Forsythia viridissima, Mentha arvensis, Zingiber officinale, Schizonepeta tenuifolia, Saposhnikovia divaricata, Ephedra sinica, Rheum undulatum, Atractylodes japonica, Platycodon grandiflorum, Scutellaria baicalensis, Glycyrrhiza uralensis, Gypsum, Talcum*, and *Natrii sulfas* (Lee M.Y. et al., 2012). Among these, *Angelica gigas* has been reported to ameliorate depressive symptoms in corticosterone-treated rats (Lee B. et al., 2015), while *Paeonia lactiflora* has been shown to improve depression-related behavior in mice (Mao et al., 2008). *Gardenia jasminoides* has been found to increase brain-derived neurotrophic factor (BDNF) in the mouse hippocampus (Zhang et al., 2015). It has also been reported that baicalin, a component of *Scutellaria baicalensis*, exerts antidepressant activity by inhibiting monoamine oxidase (MAO)-A and -B in the rat brain (Zhu et al., 2006) and that liquiritin, a component of *Glycyrrhiza uralensis*, has antidepressant effects on rats with chronic stress-induced depression (Zhao et al., 2008). However, the therapeutic potential of BTS in the treatment of depression has not yet been investigated. In this study, we investigated the antidepressant and anti-neuroinflammatory effects of BTS in an animal model of reserpine-induced depression and *in vitro* model of lipopolysaccharide (LPS)-stimulated BV2 microglia.

## Methods

### Preparation of BTS

BTS was purchased from Hanpoong Pharm and Foods Co., Ltd. (Jeonju, Korea). The plant materials were authenticated by Dr. Goya Choi (Herbal Medicine Resources Research Center, Korea Institute of Oriental Medicine, Naju, Korea) based on their morphological characteristics. The voucher specimens were deposited in the herbarium of Herbal Medicine Resources Research Center, Korea Institute of Oriental Medicine. Assurance of quality control for all the materials was validated according to the Korean Herbal Pharmacopoeia (Korea Food and Drug Administration, 2002). All the botanical names are checked using www.theplantlist.org and listed in **Supplementary Table 1**. The BTS herb sample, containing *Angelica gigas, Paeonia lactiflora, Cnidium officinale, Gardenia jasminoides, Forsythia viridissima, Mentha arvensis, Zingiber officinale, Schizonepeta tenuifolia, Saposhnikovia divaricata, Ephedra sinica, Rheum undulatum, Natrii sulfas, Atractylodes japonica, Platycodon grandiflorum, Scutellaria baicalensis, Glycyrrhiza uralensis, Talcum*, and *Gypsum* at ratios of 1:1:1:1:1:1:1:1:1:1:1.25:1.25:1.675:1.675:1.675: 1.675:1.675:2.5 (total weight = 2.421 kg) was extracted in boiling water for 3 h. The BTS extract was then filtered and concentrated under a vacuum. The yield of the dried extract was approximately 12.97%. The extract was stored at –80°C and dissolved in phosphate-buffered saline (PBS) before use.

To standardize the BTS, gallic acid, geniposide, albiflorin, paeoniflorin, liquiritin apioside, liquiritin, nodakenin, benzoic acid, baicalin, wogonoside, and glycyrrhizin were used as markers and quantified. Quantitative and qualitative analysis of the 11 marker compounds in BTS was conducted using an optimized HPLC-PDA method. Each component in BTS was identified by comparing its retention time and UV spectrum with those of each reference standard. The retention times and amounts of the 11 marker compounds in BTS are shown in **Supplementary Figure 1**.

### Animal Experiments

**Figure 1:**
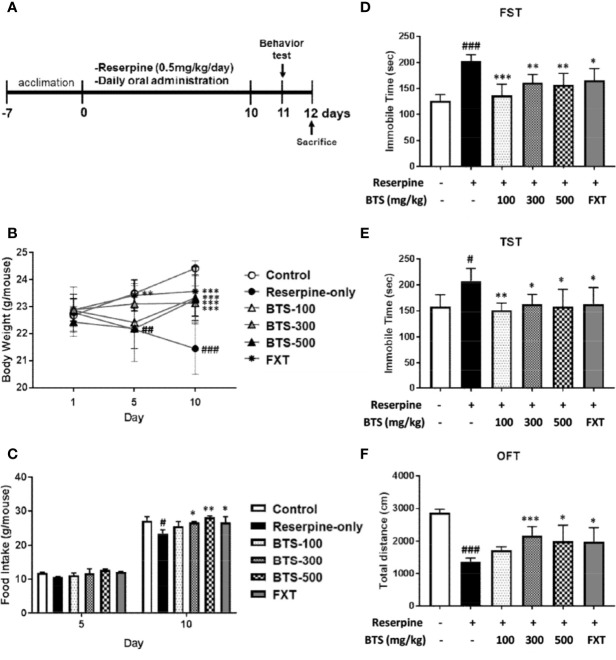
Effects of BTS on development and depression-related behaviors of reserpine-induced depression in mice. Mice were orally administered vehicle (PBS), BTS (100, 300, or 500 mg/kg), or fluoxetine (FXT, 20 mg/kg) for the indicated durations on a daily base. **(A)** Reserpine treatment and oral administration schedules for the behavior experiments. **(B)** Body weight and **(C)** food intake were measured on the indicated days. **(D)** Immobility time in the forced swimming test (FST) and **(E)** the tail suspension test (TST) and **(F)** total travel distance in the open field test (OFT), measured on day 11. Data are mean ± SD values (n = 6, one-way ANOVA: ^#^p < 0.05, ^##^p < 0.01, ^###^p < 0.001 vs. control; *p < 0.05, **p < 0.01, ***p < 0.001 vs. reserpine-only).

Seven-week-old male C57BL/6 mice were purchased from Daehan Biolink Co. (Chungbuk, Korea). Animal experiments were performed in accordance with the National Institutes of Health (NIH) Guide for the Care and Use of Laboratory Animals and approved by the Korea Institute of Oriental Medicine Institutional Animal Care and Use Committee (written approval number: 17-049). The mice were housed in polypropylene cages maintained under standard conditions at a 12-h light/dark cycle, 24 ± 0.5°C, and 55 ± 5% humidity, with standard food and water provided. To induce depression, the mice were acclimated for 1 week before receiving intraperitoneal (IP) injections of reserpine (0.5 mg/kg in PBS containing 0.1% dimethyl sulfoxide and 0.3% Tween-80) (Sigma-Aldrich, St. Louis, MO, USA) once per day for 10 days. Control mice were injected with PBS containing 0.1% dimethyl sulfoxide and 0.3% Tween-80 without reserpine. The reserpine-induced mice were randomly divided into five groups (n = 6 per group) and orally treated with PBS (reserpine-only group), 20 mg/kg fluoxetine (FXT) (Sigma-Aldrich), or 100, 300, or 500 mg/kg BTS. The control mice were administered oral doses of PBS. The experimental schemes, including reserpine induction and administration schedules, are presented in **Figure 1A**.

### Body Weight Changes and Food Intake

Body weight was measured on days 1, 5, and 10. Food intake was estimated as the difference between the amount of food remaining in the feeder on day 10 from that provided on day 1 (Park et al., 2017).

### Behavioral Tests

For the open field test (OFT), an open field arena (30 × 30 cm) was constructed from acrylic sheets, and each mouse was placed in the center of the field. The mice were individually transferred to the test field, and their behaviors were recorded for 10 min. The recordings were analyzed using video tracking software (EthoVision XT 9.0, Noldus Information Technology, Wageningen, Netherlands), as described by Deussing (Deussing, 2006). For the tail suspension test (TST), mice that were both acoustically and visually isolated were suspended 50 cm above the floor by adhesive tape placed approximately 1 cm from the tip of the tail. Immobility time was recorded during the last 4 min of a 6-min session using video tracking software (SMART 3.0; Panlab S.I., Barcelona, Spain). For the forced swimming test (FST), mice underwent a 15-min swimming session the day before the test. On the test day, mice were individually forced to swim in a cylinder (45-cm height, 20-cm diameter) containing tap water (25 ± 2°C, 25-cm depth) (Choi et al., 2017a). Total immobility time was measured during the last 4 min of a 6-min swim using the same video tracking software as in the TST. Mice underwent behavioral testing in the following order, with a 6-h interval between each experiment: OFT, TST, FST.

### Electrospray Ionization Mass Spectrometry

Whole brain samples were homogenized in 1 ml distilled water by a homogenizer (POLYTRON PT 2500 E, Luzern, Switzerland) and lysed at 145 rpm for 120 s at ambient temperature. The sample was diluted 1:9 (v/v) with methanol and centrifuged at 13000×*g* for 10 min at ambient temperature. Five microliters of upper clear was injected into the liquid chromatograph-tandem mass spectrometer (LC/MS/MS). Standards for dopamine, serotonin, and norepinephrine were purchased from Sigma-Aldrich, and standard solutions were prepared in analytical-grade methanol (Merck KGaA). An optimized multiple reaction monitoring (MRM) method was developed using ultra-performance liquid chromatography (UPLC) coupled with tandem mass spectrometry (MS/MS). A UPLC system (Acquity system, Waters) was coupled to a Xevo TQ-S triple quadrupole mass spectrometer (Waters). Chromatographic separations were carried out using a reverse-phase hybrid column (Synergi Hydro-RP, 4 μm; Phenomenex, Torrance, CA, USA) maintained at 30°C. Serotonin, dopamine, and norepinephrine were separated using gradient elution with a flow rate of 0.2 ml/min. Mobile phase solvent A was 0.1% formic acid (Sigma-Aldrich) in water, and solvent B was 0.1% formic acid in acetonitrile. The samples were eluted according to a linear gradient from 3% to 100% solvent B for 10 min. Ions were generated in positive ionization mode using an electrospray ionization interface. MS/MS analysis was performed using MRM mode by monitoring the transition pairs of *m/z* 177.2 → 160.0 for serotonin, *m/z* 154.1 → 137.0 for dopamine, and *m/z* 170.1 → 152.1 for norepinephrine. The gas flow of desolvation, cone, and nebulizer were set at 650 l/h, 150 l/h, and 7 bar, respectively.

### Immunofluorescence

The hippocampus was frozen at –20°C and cut to a thickness of 20 μm using a Cryostat Microtome (CM 3050 S, Leica Microsystems, Wetzlar, Germany). For double immunofluorescence staining, the tissue sections were incubated with antibodies specific for BDNF (Novus Biologicals, Inc., Littleton, CO, USA), phosphorylated (p)-cAMP response element-binding protein (p-CREB), and CREB (Cell Signaling Technology, Inc., Danvers, MA, USA) overnight at 4°C. Subsequently, fluorescein isothiocyanate-conjugated secondary antibody was added for 2 h, and nuclear staining was performed with 4′,6-diamidino-2-phenylindole (DAPI). All tissue samples were observed with an Eclipse T*i*-E inverted fluorescent microscope (Nikon, Tokyo, Japan).

### Cell Culture

LPS and FXT were purchased from Sigma-Aldrich. Tin protoporphyrin IX (SnPP), a heme oxygenase-1 (HO-1) inhibitor, and cobalt protoporphyrin IX (CoPP), a HO-1 inducer, were purchased from Santa Cruz Biotechnology (Santa Cruz, CA, USA). Murine BV-2 microglial cells were obtained from Dr. S.W. Chae (Korea Institute of Oriental Medicine) and cultured in Dulbecco’s modified Eagle’s medium (DMEM; Lonza, Walkersville, MD, USA) supplemented with 10% fetal bovine serum (FBS; Gibco, Gaithersburg, MD, USA) and 100 μg/ml penicillin-streptomycin (Gibco) at 37°C in a humidified atmosphere containing 5% CO_2_.

### Nitric Oxide Determination

BV2 cells (2 × 10^5^/ml) were pretreated with BTS and FXT for 1 h and then stimulated with LPS (100 ng/ml) for 24 h. The levels of nitric oxide (NO) in the culture supernatant were determined using an NO detection kit (iNtRON Biotechnology, Seongnam, Korea), according to the manufacturer’s instructions.

### Enzyme-Linked Immunosorbent Assay (ELISA)

Mouse blood was collected into heparin-coated tubes under anesthesia with Zoletil (25 mg/kg, Zoletil 50; Virbac, Cedex, France). For plasma collection, the samples were centrifuged for 10 min at 3,000 rpm at 4°C, and the supernatant was carefully transferred to a new tube. The plasma was stored at −80°C before use. Plasma levels of mouse corticosterone (Cayman Chemical Company, Ann Arbor, MI, USA) were determined using ELISA kits, according to the manufacturer’s protocols.

BV2 cells (2 × 10^5^/ml) were pretreated with BTS or FXT for 1 h and then stimulated with LPS (100 ng/ml) for 16 h. The levels of IL-6, IL-1β, TNF-α, and IL-10 in the culture supernatant were determined using a commercially available ELISA kit (R&D systems, Minneapolis, MN, USA), according to the manufacturer’s protocols.

### Real-Time PCR

Total RNA was isolated using TRIzol reagent (Invitrogen, Carlsbad, CA, USA), and cDNA synthesis was performed using the PrimeScript^™^ RT reagent kit (TaKaRa, Shiga, Japan). The mRNA levels of *Il1b, Il6, Tnfa, Nos2, Cox2, Hmox1*, and glyceraldehyde-3 phosphate dehydrogenase (*Gapdh*) were quantified using a 7500 real-time PCR system (Applied Biosystems, Foster City, CA, USA) with Power SYBR^®^ Green PCR Master Mix (Applied Biosystems) (Park et al., 2015). Primer sequences are listed in **Table 1**.

**Table 1:** Sequences of the real-time PCR primers.

Mouse gene	Sequence
*Il1b*	forward, 5′- GCTGAAAGCTCTCCACCTCA -3′ reverse, 5′- AGGCCACAGGTATTTTGTCG -3′
*Il6*	forward, 5′- GAGGATACCACTCCCAACAGACC -3′ reverse, 5′- AAGTGCATCATCGTTGTTCATACA -3′
*Tnfa*	forward, 5′- AGACCCTCACACTCAGATCATCTTC -3′ reverse, 5′- CCACTTGGTGGTTTGCTACGA -3′
*Hmox1*	forward, 5′- AGCCCCACCAAGTTCAAACA -3′ reverse, 5′- CATCACCTGCAGCTCCTCAA -3′
*Nos2*	forward, 5′- GAATCTTGGAGCGAGTTGTGGA -3′ reverse, 5′- GTGAGGGCTTGGCTGAGTGAG -3′
*Cox2*	forward, 5′- TGGGGTGATGAGCAACTATT-3 reverse, 5′- 5-AAGGAGCTCTGGGTCAAACT-3
*Gapdh*	forward, 5′- AAGGTGGTGAAGCAGGCAT -3′ reverse, 5′- GGTCCAGGGTTTCTTACTCCT -3′

### Western Blotting

Brain samples were homogenized in 1 ml of lysis buffer (Pro-Prep™; iNtRON Biotechnology) containing 1 mM phenylmethylsulfonyl fluoride (PMSF) and 1 µg/ml protease inhibitor mixture. Whole-cell lysates were harvested using radioimmunoprecipitation assay (RIPA) buffer containing protease inhibitor cocktail (Sigma-Aldrich), 1 mM PMSF, and phosphatase inhibitor cocktail set III (Calbiochem, San Diego, CA, USA). Nuclear extracts were prepared using a nuclear extract kit (Active Motif, Carlsbad, CA, USA). Equal amounts (20 μg) of protein were separated using 10% sodium dodecyl sulfate-polyacrylamide gel electrophoresis and transferred to polyvinylidene fluoride membranes (Amersham Biosciences, Piscataway, NJ, USA), which were blocked with 5% skim milk in TBS/T (Tris-buffered saline in 0.1% TWEEN^®^ 20) buffer for 1 h. The membranes were then treated overnight with antibodies specific to iNOS, nuclear factor (NF)-κB p65, HO-1, nuclear factor erythroid 2-related factor 2 (NRF2), phospho-CREB, CREB, phospho-p38 (Thr180/Tyr182), p38, phospho-Erk (Thr202/Tyr204), Erk, phospho-JNK (Thr183/Tyr185), JNK, phospho-Akt (Ser473), Akt (Cell Signaling Technology, Inc.) and BDNF (Abcam, Cambridge, UK) at 4°C. Blots were incubated with horseradish peroxidase (HRP)-conjugated secondary antibody for 2 h at room temperature. HRP was detected using a chemiluminescent detection reagent (Amersham Biosciences). β-Actin (Sigma-Aldrich) and proliferating cell nuclear antigen (PCNA; Cell Signaling Technology, Inc.) were used as loading controls. Chemiluminescence was visualized using an LAS-3000 LuminoImage analyzer (Fujifilm, Tokyo, Japan) (Park et al., 2013).

### Statistical Analysis

All data are expressed as mean ± standard deviation (SD). One-way analysis of variance (ANOVA) was performed using GraphPad Prism version 7 (GraphPad Software Inc., San Diego, CA, USA) to assess between-group differences. Multiple group comparisons were performed using one-way ANOVA, followed by post-hoc Tukey tests. Differences at p < 0.05 were considered statistically significant.

## Results

### Effects of BTS on Body Weight Changes and Food Intake

Mean body weight did not significantly differ among the groups on day 1. After reserpine injection, reserpine-only mice demonstrated a decrease in body weight, observed on days 5 and 10, compared to control mice; however, oral BTS administration suppressed the body weight decrease on day 10 (BTS 100: 23.33 ± 0.86 g, BTS 300: 23.13 ± 0.64 g, and BTS 500: 23.27 ± 0.9 g, all p < 0.001; **Figure 1B**). FXT administration (20 mg/kg) significantly increased body weight on days 5 (FXT: 23.42 ± 0.57 g, p < 0.01; **Figure 1B**) and 10 (FXT: 23.57 ± 0.92 g, p < 0.001; **Figure 1B**). Regarding food intake, reserpine-treated mice showed a significant reduction in food intake compared to that of control mice on day 10 (23.51 ± 1.09 g, p < 0.05). However, BTS and FXT treatments counteracted the reduction in food intake on day 10 (BTS 300: 26.79 ± 0.21 g, p < 0.01; BTS 500: 28.22 ± 0.4 g, p < 0.05; FXT: 26.79 ± 1.6 g, p < 0.05; **Figure 1C**). These results suggest that BTS counteracted weight loss and food intake reduction in mice with reserpine-induced depression.

### Effects of BTS on Behavioral Tests

In the FST [F (5, 30) = 12.99, p < 0.001; **Figure 1D**], we measured immobilization time on day 11. As expected, immobilization time significantly increased in reserpine-treated mice compared with that in control mice (203.28 ± 11.58 s, p < 0.001), whereas in BTS-treated mice, immobilization time was markedly reduced compared with that in reserpine-treated mice (BTS 100: 136.46 ± 21.7 s, p < 0.001; BTS 300: 161.66 ± 15.24 s, p < 0.01; BTS 500: 156.93 ± 22.16 s, p < 0.01; **Figure 1D**). FXT treatment also significantly decreased the immobilization time (FXT: 165.63 ± 22.54 s, p < 0.05). In the TST [F (5, 30) = 3.867, p < 0.01; **Figure 1E**), the immobilization time decreased in BTS-treated mice (BTS 100: 151.37 ± 14.01 s, p < 0.01; BTS 300: 162.57 ± 19.19 s, p < 0.05; BTS 500: 158.23 ± 33.32 s, p < 0.05) compared to that in reserpine-treated mice (**Figure 1E**). Similarly, in the OFT [F (5, 30) = 3.867, p < 0.01; **Figure 1F**], the travel distance increased in BTS-treated mice (BTS 300: 2161.26 ± 281.19 cm, p < 0.001; BTS 500: 1997.62 ± 486.63 cm, p < 0.05) compared to that in reserpine-treated mice (**Figure 1F**). FXT treatment resulted in effects similar to those of BTS. These results suggest that BTS might control depressive-like behaviors in mice with reserpine-induced depression.

### Effects of BTS on Mood-Related Hormones in Reserpine-Treated Mice

**Figure 2:**
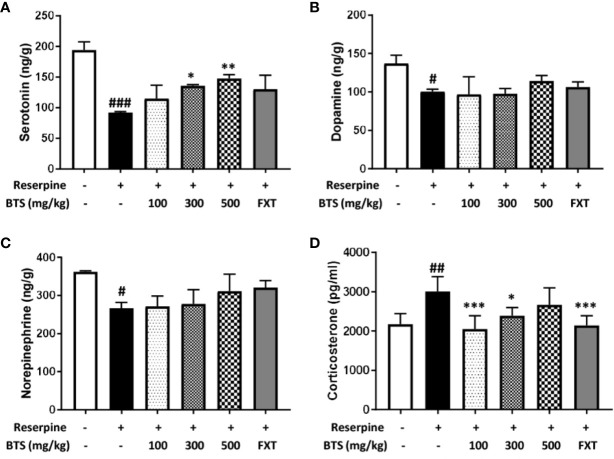
Effects of BTS on monoamines and serum levels of corticosterone in reserpine-induced depression in mice. **(A)** Serotonin, **(B)** dopamine, and **(C)** norepinephrine levels in the brain were measured by electrospray ionization mass spectrometry. **(D)** Serum levels of corticosterone were determined by ELISA. The data represent the mean ± SD of triplicate determinations (n = 6, one-way ANOVA: ^#^p < 0.05, ^##^p < 0.01, ^###^p < 0.001 vs. control; *p < 0.05, **p < 0.01, ***p < 0.001 vs. reserpine-only).

Serotonin levels in the brain, an indicator of mood disorders (Martinowich and Lu, 2008), were significantly decreased in reserpine-treated mice, but oral BTS and FXT administration reversed this decrease (**Figure 2A**). Plasma levels of the stress hormone corticosterone (Liu et al., 2010) significantly increased in reserpine-treated mice but decreased in BTS- and FXT-treated mice (**Figure 2D**). These data suggest that BTS might have effects on mood-related hormones in reserpine-induced depression in mice. Notably, dopamine and norepinephrine levels in the brain were not significantly increased in BTS mice (**Figures 2B, C**).

### Effects of BTS on BDNF and p-CREB Expression in the Brain

**Figure 3:**
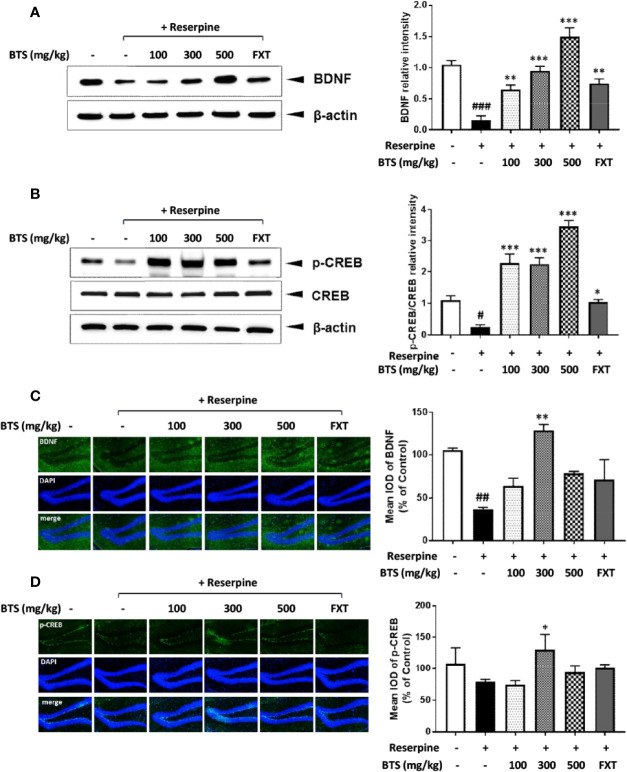
Effects of BTS on BDNF and p-CREB/CREB expression and histology in the hippocampus of reserpine-induced depressed mice. Isolated hippocampus lysates were analyzed by western blotting using **(A)** BDNF and **(B)** p-CREB/CREB antibodies. β-actin was used as the loading control. Frozen hippocampus sections were analyzed by immunofluorescence using **(C)** BDNF and **(D)** p-CREB antibodies. DAPI was used as the loading control. The presented data are representative of three independent experiments. The data represent the mean ± SD (one-way ANOVA: ^#^p < 0.05, ^##^p < 0.01, ^###^p < 0.001 vs. control; *p < 0.05, **p < 0.01, ***p < 0.001 vs. reserpine-only).

To understand the effects of BTS on depression-like symptoms at the molecular level, BDNF and p-CREB expression in the brain was examined by western blotting. BDNF levels dramatically decreased in reserpine-treated mice compared with those in control mice, indicating neuronal dysfunction of the brain (Lu et al., 2014); however, BTS treatment significantly enhanced BDNF levels in a dose-dependent manner in the hippocampus (**Figure 3A**). The BDNF-CREB pathway is associated with MDD (Nair and Vaidya, 2006). The level of p-CREB also decreased in the brains of reserpine-treated mice and markedly increased in BTS and FXT mice (**Figure 3B**). These results suggest that BTS might affect neuronal activity in the mouse brain in reserpine-induced depression.

To confirm the effect of BTS treatment on our reserpine-induced depression model, we observed BDNF and p-CREB expression in the hippocampus using immunofluorescence. BDNF levels were decreased in the reserpine-treated group, and this decrease was counteracted by treatment with 300 mg/kg BTS (**Figure 3C**). Likewise, the same results were obtained for p-CREB expression (**Figure 3D**). These changes were seen in the dentate gyrus of the hippocampus. These results suggest that BTS attenuates reserpine-induced depression *via* activation of the BDNF-CREB pathway.

### Effects of BTS on Pro-Inflammatory Cytokines in Reserpine-Induced Depression

**Figure 4:**
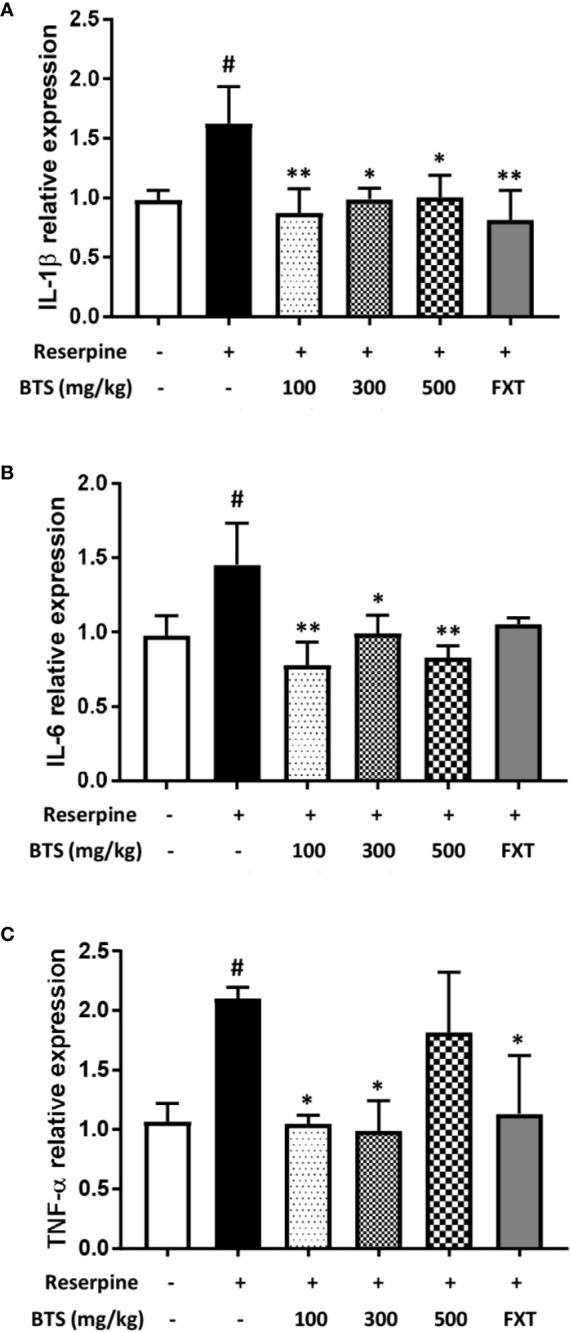
Effects of BTS on *Il1b, Il6*, and *Tnfa* mRNA expression in the hippocampus of mice with reserpine-induced depression. **(A)** mRNA levels of *Il1b*, **(B)** *Il6*, and **(C)** *Tnfa* were determined by quantitative real-time PCR. The data represent the mean ± SD of triplicate determinations (n = 6, one-way ANOVA: ^#^p < 0.05 vs. control; *p < 0.05, **p < 0.01 vs. reserpine-only).

Pro-inflammatory cytokines such as IL-1β, IL-6, and TNF-α, which are released through the activation of the neuroendocrine system, contribute to neuroinflammation (Krishnan and Nestler, 2008). *Il1b, Il6*, and *Tnfa* mRNA levels in the hippocampus were significantly increased in reserpine-treated mice but were reduced in BTS and FXT mice (**Figure 4**). These data suggest that BTS might be involved in various immune reactions in the central nervous system.

### Effects of BTS on NO Production and Nos2 and Cox2 Expression in LPS-Stimulated BV2 Microglia

**Figure 5:**
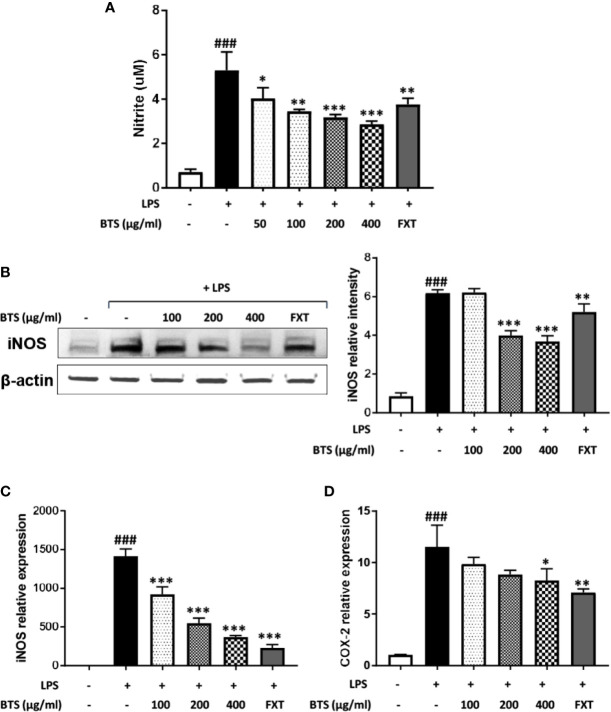
Effect of BTS on NO production and iNOS and COX-2 expression in BV2 cells. BV2 cells were pretreated with BTS for 1 h and then stimulated with LPS (100 ng/ml) for 24 h. **(A)** The levels of NO in the cell culture supernatant were measured by NO detection kit. **(B)** The level of iNOS was determined by western blotting. β-actin was used as a loading control. The data represent three independent experiments. BV2 cells were pretreated with BTS for 1 h and then stimulated with LPS (100 ng/ml) for 6 h. Levels of **(C)** *Nos2* and **(D)** *Cox2* mRNAs were determined by real-time PCR. *Gapdh* was used as a loading control. The data represent the mean ± SD of triplicate determinations (one-way ANOVA: ^###^p < 0.001 vs. untreated control; *p < 0.05, **p < 0.01, ***p < 0.001 vs. LPS-treated control).

In BV2 microglia, we first performed a 3-(4,5-dimethylthiazol-2-yl)-5-(3-carboxymethoxyphenyl)-2-(4-sulfophenyl)-2H–tetrazolium (MTS) assay to determine the cytotoxicity of BTS. The result of this assay demonstrated that BTS is not cytotoxic at concentrations of up to 800 μg/ml (data not shown). To investigate the effects of BTS on NO production, BV2 cells were treated with LPS (100 ng/ml) in presence or absence of BTS. The production of NO was increased up to sevenfold following LPS stimulation, and treatment with BTS at 50–400 μg/ml significantly blocked this a dose-dependent manner (**Figure 5A**). iNOS protein levels were also dramatically increased in LPS-treated cells; similar to the results for NO, treatment with BTS at 200 or 400 μg/ml suppressed this increase (**Figure 5B**). Further, as shown in **Figures 5C, D**, *Nos2* and *Cox2* mRNA levels were significantly increased by LPS stimulation. BTS at 400 μg/ml significantly suppressed these increases in *Nos2* and *Cox2* gene expression. Furthermore, FXT at 10 μM, serving as a positive control, also had inhibitory effects on NO production and *Nos2* and *Cox2* gene expression. These data suggest that BTS may inhibit NO production through the downregulation of *Nos2* and *Cox2*.

### Effects of BTS on Pro-Inflammatory Cytokine Production and mRNA Expression in LPS-Stimulated BV2 Microglia

**Figure 6:**
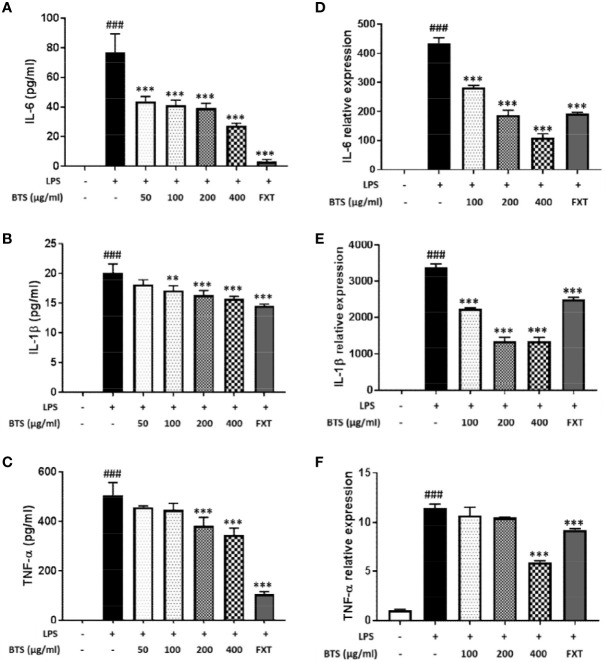
Effect of BTS on expression of pro-inflammatory cytokines in BV2 cells. BV2 cells were pretreated with BTS for 1 h and then stimulated with LPS (100 ng/ml) for 6 h. mRNA expression levels of **(A)** *Il6*, **(B)** *Il1b*, and **(C)** *Tnfa* were determined by real-time PCR. *Gapdh* was used as a loading control. BV2 cells were pretreated with BTS for 1 h and then stimulated with LPS (100 ng/ml) for 16 h. Levels of **(D)** IL-6, **(E)** IL-1β, and **(F)** TNF-α in the cell culture supernatant were measured by ELISA. The data represent the mean ± SD of triplicate determinations (one-way ANOVA: ^###^p < 0.001 vs. untreated control; **p < 0.01, ***p < 0.001 vs. LPS-treated control).

LPS-stimulated microglia produce high levels of cytokines, such as IL-6, IL-1β, and TNF-α. The production of IL-6, IL-1β, and TNF-α was markedly increased in LPS-treated control cells; however, pretreatment with BTS significantly inhibited increases in cytokine levels in a dose-dependent manner (**Figures 6A–C**). Furthermore, mRNA levels of *Il6, Il1b*, and *Tnfa* were dramatically increased by LPS stimulation and significantly decreased by BTS in a dose-dependent manner (**Figures 6D–F**). FXT at 10 μM also showed inhibitory effects on the production and mRNA levels of these cytokines.

### Effects of BTS on Activation of Mitogen-Activated Protein Kinase (MAPK), Phosphatidylinositol 3-Kinase (PI3K)/Akt, and NF-κB Inflammatory Pathways in LPS-Stimulated BV2 Microglia

**Figure 7:**
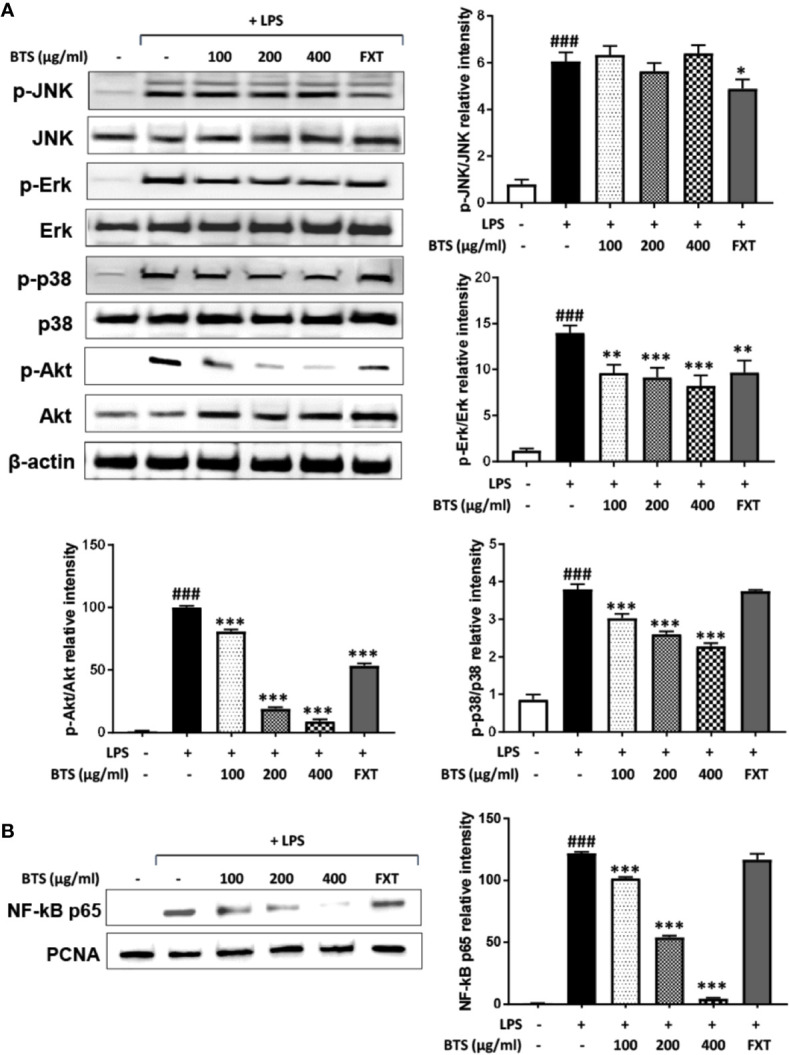
Effect of BTS on the phosphorylation of MAPKs and Akt and NF-κB activity in BV2 cells. **(A)** BV2 cells were pretreated with BTS for 1 h and then stimulated with LPS (100 ng/ml) for 15 min. Levels of p-JNK, JNK, p-Erk, Erk, p-p38, p38, p-Akt, and Akt were determined by western blotting. β-actin was used as a loading control. **(B)** BV2 cells were pretreated with BTS for 1 h and then stimulated with LPS (100 ng/ml) for 1 h. Nuclear extracts were analyzed by western blotting using NF-κB p65 antibody. PCNA was used as a loading control. The data represent three independent experiments. The data are expressed as the mean ± SD (one-way ANOVA: ^###^p < 0.001 vs. untreated control; *p < 0.05, **p < 0.01, ***p < 0.001 vs. LPS-treated control).

MAPKs, PI3K/Akt, and NF-κB play important roles in signaling pathways that induce a neuroinflammatory response in microglia (Park et al., 2014). Given this, the effects of BTS on the MAPK, PI3K/Akt, and NF-κB pathways were examined. As shown in **Figure 7A**, levels of p-Erk in control cells were minimal, whereas treatment with LPS dramatically increased these levels. Additionally, BTS reduced p-Erk levels, while total Erk levels were unchanged. Levels of p-p38 were dramatically increased after LPS, and this was reversed by BTS treatment. However, levels of p-JNK were unchanged by BTS treatment. Furthermore, the phosphorylation of Akt was significantly increased by LPS treatment, while total Akt was not affected. Upon BTS treatment, levels of p-Akt were significantly reduced. NF-κB is a key transcription factor that modulates iNOS and pro-inflammatory cytokine gene expression in microglia. Western blotting analyses using nuclear extracts from treated and control BV2 cells indicated that expression of p65, a component of NF-κB, significantly increased upon LPS stimulation. Pretreatment with BTS significantly inhibited expression of this factor in the nucleus (**Figure 7B**). These data suggest that BTS may interfere with Erk, p38, and Akt to facilitate altered NF-κB pathway signaling and inhibit neuronal pro-inflammatory responses to LPS stimulation.

### Effects of BTS on IL-10 and HO-1 Expression *via* Upregulation of NRF2/CREB Pathway in BV2 Microglia

**Figure 8:**
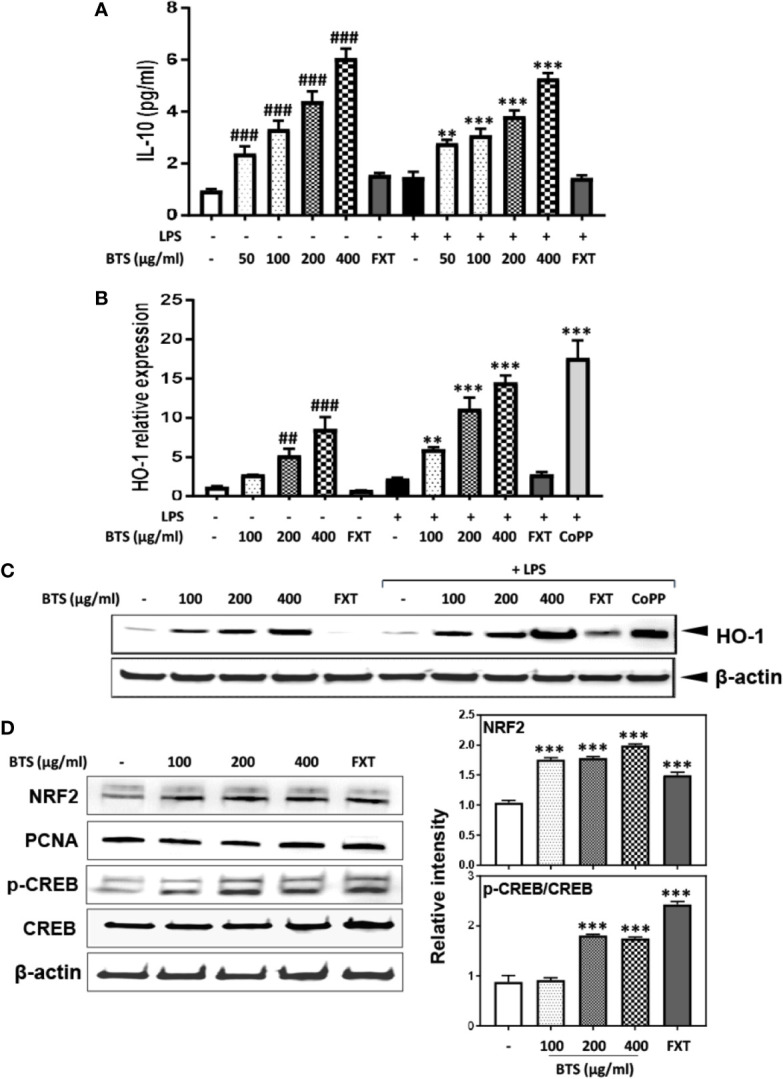
Effect of BTS on IL-10 production and HO-1 expression in BV2 cells. **(A)** BV2 cells were pretreated with BTS for 1 h and then stimulated with LPS (100 ng/ml) for 16 h. Levels of IL-10 in the cell culture supernatant were measured by ELISA. **(B)** BV2 cells were pretreated with BTS for 1 h and then stimulated with LPS (100 ng/ml) for 6 h. *Hmox1* mRNA levels were determined by real-time PCR. *Gapdh* was used as a loading control. **(C)** BV2 cells were pretreated with BTS for 1 h and then stimulated with LPS (100 ng/ml) for 12 h. The level of HO-1 was determined by western blotting. β-actin was used as a loading control. **(D)** BV2 cells were pretreated with BTS for 6 h, and nuclear extracts were analyzed by western blotting using an NRF2 antibody. PCNA was used as a loading control. BV2 cells were pretreated with BTS for 30 min, and levels of p-CREB and CREB were determined by western blotting. β-actin was used as a loading control. The data represent three independent experiments. The data are expressed as the mean ± SD of triplicate determinations (one-way ANOVA: ^##^p < 0.01, ^###^p < 0.001 vs. untreated control; **p < 0.01, ***p < 0.001 vs. LPS-treated control).

IL-10 and HO-1 act as anti-inflammatory modulators *via* the upregulation of the NRF2/CREB pathway in microglia (Lee E.J. et al., 2015). Given this, we examined the effect of BTS on IL-10 and HO-1 expression in LPS-treated and control BV2 cells. Levels of IL-10 production increased in a dose-dependent fashion following BTS treatment. LPS-treated cells produced similar levels of IL-10 as those treated with BTS only (**Figure 8A**). Next, we examined the mRNA and protein expression of HO-1, both of which were significantly increased in LPS-treated control cells. BTS and LPS treatment increased HO-1 expression over levels in BTS-only-treated cells (**Figures 8B, C**). CoPP at 20 μM was used as a positive control to induce HO-1 expression (**Figures 8B, C**). Furthermore, BTS increased the nuclear translocation of NRF2 and p-CREB, which act as upstream modulators of HO-1 expression (**Figure 8D**). These data suggest that BTS might upregulate HO-1 *via* the NRF2/CREB pathway to induce neuroprotective effects in BV2 microglia.

### HO-1 Mediates the Effects of BTS on NO Production and Pro-Inflammatory Cytokine mRNA Expression in BV2 Microglia

**Figure 9:**
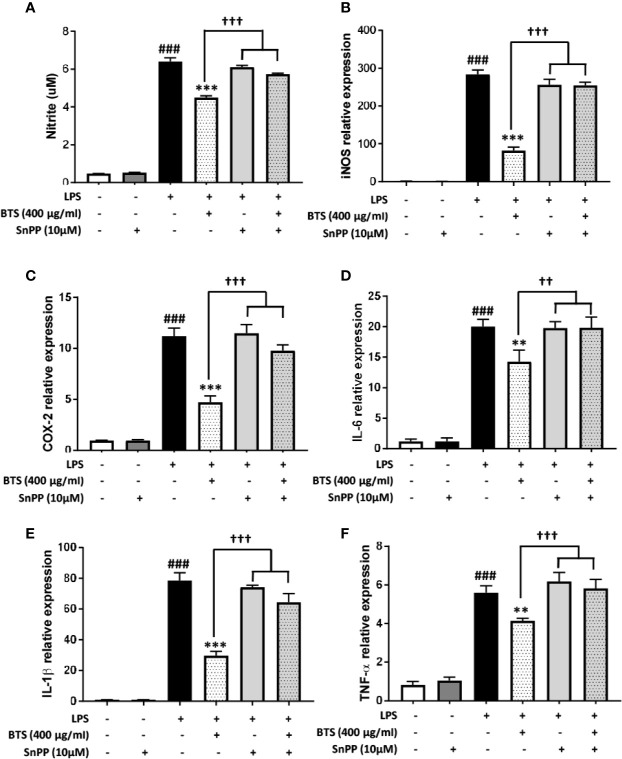
HO-1 mediates the effect of BTS on NO production and mRNA expression of pro-inflammatory cytokines in BV2 cells. **(A)** BV2 cells were pretreated with BTS for 1 h in the presence or absence of SnPP (10 μM) and then stimulated with LPS (100 ng/ml) for 24 h. Levels of NO in the cell culture supernatant were measured by NO detection kit. **(B–F)** BV2 cells were pretreated with BTS for 1 h in the presence or absence of SnPP (10 μM) and then stimulated with LPS (100 ng/ml) for 6 h. Levels of **(B)** *Nos2*, **(C)** *Cox2*, **(D)** *Il6*, **(E)** *Il1b*, and **(F)** *Tnfa* mRNAs were determined by real-time PCR. *Gapdh* was used as a loading control. The data represent the mean ± SD of triplicate determinations (one-way ANOVA: ^###^p < 0.001 vs. untreated control; **p < 0.01, ***p < 0.001 vs. LPS-treated control; ^††^p < 0.01, ^†††^p < 0.001 vs. LPS+BTS).

To confirm the mechanism by which BTS affects pro-inflammatory signaling pathways, we examined whether HO-1 mediates the effects of BTS on NO production and pro-inflammatory cytokine gene expression. This was assessed by co-treating cells with SnPP, an inhibitor of HO-1 activity. We found that BTS significantly reduced NO production and *Nos2*, *Cox2*, and pro-inflammatory cytokine gene expression levels. However, SnPP reversed the inhibitory effects of BTS on NO production and *Nos2*, *Cox2*, and pro-inflammatory cytokine mRNA levels (**Figures 9A–F**). Collectively, these results suggest that BTS acts as an anti-neuroinflammatory and neuroprotective agent in BV2 microglia *via* the upregulation of HO-1.

## Discussion

Although the pathophysiology of depression has not yet been fully elucidated, the typically assumed molecular biologic causes include a lack of monoamines such as serotonin, noradrenaline, and dopamine; hyperactivity of the hypothalamic-pituitary-adrenal (HPA) axis (Stetler and Miller, 2011); neuroinflammation (Schiepers et al., 2005); BDNF dysfunction; decreased gamma-aminobutyric acid (GABA) activity; and glutamate system dysfunction (Maletic et al., 2007; Hasler, 2010).

In our experiments, we used a model of depression induced by reserpine-mediated depletion of monoamines in the brains of mice. Reserpine has been shown to block amine storage processes, thereby leading to increased hippocampal excitability and blood corticoid levels, which are related to 5-hydroxytryptamine (5-HT) changes in the brain (Revzin et al., 1962). The reserpine model represents the first neurological model of depression and has been used in many depression-related studies since the 1960s (Schildkraut, 1965). FST, TST, and OFT are typical behavioral tests that show animal anxiety and depression-related patterns (Deussing, 2006). They are mainly used in screening tests for antidepressants. Mice with reserpine-induced depression have been reported to show anxiety and depression-related behaviors such as increased immobility time and decreased total travel distance in these behavioral tests compared with those of normal mice (Tian et al., 2010). They also exhibit increases in corticosterone blood levels (Revzin et al., 1962) and pro-inflammatory cytokines in the brain (Szelenyi and Vizi, 2007). In our experiments, BTS-treated mice showed significant decreases in immobility time in the FST and TST and an increase in total travel distance in the OFT compared with those of reserpine-treated mice. Moreover, the serotonin level was increased and corticosterone level was decreased in BTS-treated mice compared with those in reserpine-treated mice.

BDNF and CREB have been reported to be involved in neuronal differentiation and survival, as well as in synaptic plasticity associated with learning and memory in various nervous system disorders, including depression (Nair and Vaidya, 2006). Our reserpine-induced mouse model showed depressive symptoms as well as decreased BDNF levels and neurogenesis in the hippocampus. Western blotting and immunofluorescence analysis showed that the expression of BDNF and p-CREB in the hippocampus was increased in BTS-treated mice compared to those in reserpine-treated mice. In addition, in our reserpine-induced animal model of depression, levels of IL-1β, IL-6, and TNF-α were significantly higher than those in control mice. The extracellular catecholamine level balance is one of the key modulators of inflammatory mediator production (Huang et al., 2004; Szelenyi and Vizi, 2007). By measuring the mRNA levels of pro-inflammatory cytokine genes in the hippocampus, we confirmed that *Il1b, Il6*, and *Tnfa* mRNA levels in BTS mice were reduced compared to those in reserpine-treated mice.

Microglia are involved in innate immunity and regulate cytokine levels and inflammatory processes in the brain (Harry and Kraft, 2008). When activated by infection or tissue damage, microglia produce inflammatory factors, including pro-inflammatory cytokines and reactive oxygen species, which can cause neuronal toxicity and degeneration (Graeber and Streit, 2010). To complement our *in vivo* results, we performed *in vitro* analyses to better understand the mechanisms underlying the anti-neuroinflammatory effects of BTS. LPS stimulation of microglia results in activation of Toll-like receptors (TLRs), phosphorylation of MAPKs, and translocation of NF-κB p65, an inflammatory transcription factor, into the nucleus *via* PI3K/Akt, where it increases the production of various pro-inflammatory cytokines and reactive oxygen species (Guo et al., 2016; Choi et al., 2017b). In contrast, an increase in neuroprotective factors, such as NRF2, CREB, and HO-1 can produce the anti-inflammatory cytokine IL-10 and thus inhibit neuroinflammation.

In the present study, we found that BTS inhibited NO production by suppressing the expression of iNOS and COX-2, key enzymes in NO production. In addition, production and mRNA expression levels of TNF-α, IL-1β, and IL-6 were inhibited by BTS *via* suppression of the activation of NF-κB and the phosphorylation of Erk and p-38 in specific MAPK pathways and of Akt in LPS-stimulated BV2 cells. Our data clearly demonstrated that BTS significantly increased HO-1 expression *via* increases in the nuclear translocation of NRF2 and phosphorylation of CREB. Furthermore, levels of IL-10, an anti-inflammatory cytokine (Bozic et al., 2015), were increased by BTS in both the presence and absence of LPS.

Antidepressants, which are widely used in the treatment of depression, are drugs that target monoamines (Mahar et al., 2014). However, these drugs have many side effects, are difficult to use for long periods, and are not effective in the 30%–40% of cases with treatment-resistant depression (Kornstein and Schneider, 2001). In addition, although the use of antipsychotic drugs and mood stabilizers has enhanced the therapeutic response in treatment-resistant depression, their efficacy is still limited (Nierenberg et al., 2007). Therefore, new natural medicines that are safe and effective have recently been attracting attention (Grosso, 2016). Several studies reported that oxidative stress causes neuroinflammation and consequent detrimental effects in major depression (Krishnan and Nestler, 2008; Lang and Borgwardt, 2013; Bakunina et al., 2015). In addition, inflammatory factors, produced during neuroinflammation, affected glutamate and monoamine neurotransmission, glucocorticoid receptor resistance, and hippocampal neurogenesis (Krishnadas and Cavanagh, 2012; Zhang et al., 2018). Therefore, we explored the antidepressant effects of BTS in an *in vivo* reserpine-induced depression model. We demonstrate that indeed neuroinflammation is one of the factors contributing to depression. Furthermore, we also show that the antidepressant effect of BTS is probably the consequence of its action in microglia as an anti-neuroinflammatory agent. Toxicity studies have reported that BTS is safe regardless of sex at concentrations of up to 2000 mg/kg/day, and standardization results in accordance with MFDS specifications have already been reported (Lee M.Y. et al., 2012). Thus, BTS is a promising candidate for the treatment of depression. In summary, our data show that BTS is effective against reserpine-induced depression in preclinical models. Mechanistically, as observed *in vitro* after LPS stimulation, BTS exerts anti-neuroinflammatory and neuroprotective effects through the upregulation of HO-1 or IL-10. Our research can provide a basis for the clinical application of BTS for managing conditions such as major depression by targeting an unusual depression mechanism, neuroinflammation in the future. We can further expect clinical effective minimum dose of BTS or consequently decrease of the associated adverse side effects when taken long-term. This hypothesis should be tested and then translated to the clinical context, provided the results are promising.

## Conclusions

Taken together, our results show that administration of BTS can induce an antidepressant-like effect in reserpine-induced depression. Furthermore, our results suggest that BTS may act as a neuroprotective agent by downregulating neuroinflammation in activated, cultured microglia. Specifically, since BTS is composed of several herbs, our results also suggest that a multi-targeted approach could improve treatment for depression. Further studies will be needed at the molecular level to investigate the regulatory effects of BTS on neuroinflammation and HPA-axis hyperactivity.

## Data Availability Statement

All datasets generated for this study are included in the article/**Supplementary Material**.

## Ethics Statement

Animal experiments were performed in accordance with the National Institutes of Health (NIH) Guide for the Care and Use of Laboratory Animals and approved by the Korea Institute of Oriental Medicine Institutional Animal Care and Use Committee (written approval number: 17-049).

## Author Contributions

B-KP performed experiments, analyzed data, and wrote the paper. NK performed experiments according to the reviewer’s suggestions, analyzed data, and wrote the supplementary paper. YK performed experiments and analyzed data. CY, IJ, and JC analyzed and discussed data. C-SS and I-SJ performed the HPLC analysis. ML designed the experiments and reviewed the manuscript. All authors contributed to the article and approved the submitted version.

## Funding

This work was supported by a grant (KSN2013220) from the Korea Institute of Oriental Medicine (KIOM) and a grant from the National Research Foundation of Korea (NRF) funded by the Korean government (MSIP) (grant number: NRF-2016R1A2B4009614).

## Conflict of Interest

The authors declare that the research was conducted in the absence of any commercial or financial relationships that could be construed as a potential conflict of interest.
