# Supplementary material for: Antidepressant and Anti-Neuroinflammatory Effects of Bangpungtongsung-San
Source: Front Pharmacol. 2020 Jul 10;11:958. doi: 10.3389/fphar.2020.00958 (PMC7366903; doi:10.3389/fphar.2020.00958)
Supplement: Supplementary file 1 [file DataSheet_1.docx]

Supplementary Material

# Supplementary Data

## 1.1 The composition and proportion of herb in BTS

BTS was purchased from Hanpoong Pharm and Foods Co., Ltd. (Jeonju, Korea). The plant materials were authenticated by Dr. Goya Choi (Herbal Medicine Resources Research Center, Korea Institute of Oriental Medicine, Naju, Korea) based on their morphological characteristics. The voucher specimens were deposited in the herbarium of Herbal Medicine Resources Research Center, Korea Institute of Oriental Medicine. Assurance of quality control for all the materials was validated according to the Korean Herbal Pharmacopoeia (Korea Food and Drug Administration, 2002). All the botanical names are checked using [www.theplantlist.org](http://www.theplantlist.org) and listed in Supplementary Table 1.

## 1.2 High-performance liquid chromatography (HPLC) analysis of BTS

HPLC analysis of the major components in the BTS decoction was performed using a Shimadzu Prominence LC–20A system (Kyoto, Japan) equipped with a photodiode array (PDA) detector. Chromatographic data were measured, acquired, and processed using LCsolution software (Version 1.24, SP1, Kyoto, Japan). Gallic acid (PubChem CID: 370, purity 99.0%) and benzoic acid (PubChem CID: 243, purity 99.9%) were purchased from Merck KGaA (Darmstadt, Germany). Geniposide (PubChem CID: 107848, purity 98.0%), albiflorin (PubChem CID: 51346141, purity 99.8%), paeoniflorin (PubChem CID: 442534, purity 98.8%), liquiritin (PubChem CID: 503737, purity 99.6%), baicalin (PubChem CID: 64982, purity 98.0%), and glycyrrhizin (PubChem CID: 14982, purity 99.0%) were obtained from Wako (Osaka, Japan). Liquiritin apioside (PubChem CID: 10076238, purity 98.0%), nodakenin (PubChem CID: 73191, purity 99.5%), and wogonoside (PubChem CID: 3084961, purity 98.2%) were purchased from Shanghai Sunny Biotech (Shanghai, China), ChemFaces Biochemical (Wuhan, China), and Tauto Biotech (Shanghai, China), respectively. The 11 marker compounds were separated on a Waters SunFire C_18_ column (250 × 4.6 mm, 5 μm, Milford, MA, USA) and maintained at 40°C. The mobile phases consisted of water (A) and acetonitrile (J. T. Baker, Phillipsburg, NJ, USA) (B), both containing 1.0% (v/v) acetic acid (Merck KGaA). The gradient elution of the mobile phase was as follows: 5 to 70% B for 0–40 min, 70 to 100% B for 40–45 min, 100% B for 45–50 min, and 100 to 5% B for 50–55 min. The flow-rate and injection volume were 1.0 mL/min and 10 μL, respectively. The retention times and amounts of the 11 marker compounds in BTS are shown in Supplementary Figure 1.

**1.3 Primary mouse microglial (MMcg) cells and culture conditions**

Neonatal primary MMcg cells and microglial culture media were supplied as a kit (code # M8816K-10n) from Cell Applications (San Diego, CA, USA, M8816K-10n). Frozen MMcg cells were thawed and resuspended in culture medium as described in the manufacture’s guide. Cells were seeded at a density 40,000 per cm^2^ in a 24-well plate. After 24 h, the culture medium was renewed and the cells were used for experiments 3 days after initial seeding. Cells were pretreated with BTS (400 µg/mL) 1 h before LPS (100 ng/mL) exposure. The levels of NO and inflammatory cytokines like IL-1β, IL-6, and TNF-α secreted into the culture medium were determined 24 h after LPS treatment as described in the Methods section.

**1.4 HO-1 knockdown and quantitative real-time PCR**

Knockdown of endogenous HO-1 expression in BV2 cells was mediated by siRNA transfection. The siRNA targeting mouse HO-1 (sc-35555, siHO-1) and negative control siRNA (sc-37007, siCtrl) were purchased from Santa Cruz Biotechnology (Santa Cruz, CA, USA). The siRNAs were introduced into BV2 cells using the RNAiMax transfection reagent (Thermo Fisher Scientific, Carlsbad, CA, USA) and Opti-MEM transfection medium (Thermo Fisher Scientific) according to the manufacturer’s instructions. Mixtures of siHO-1 or siCtrl, and transfection reagent were prepared in a 6-well culture plate. BV2 cells (2.5 x 10^5^ cells per well) were were distributed on the siRNA/transfection reagent mixes and incubated in a humidified CO_2_ incubator at 37°C. Forty eight hours after siRNA transfection, cells were pretreated with 400 µg/mL BTS for 1 h and then exposed to 100 ng/mL LPS for 6 h (quantitative PCR) or 24 h (western blot). Quantitative real-time PCR using total RNAs and western blotting using whole cell lysates were performed as described in the Methods section.

# Supplementary Tables and Figures

## Supplementary Tables

**Supplementary Table 1. The composition and proportion of herbs in BTS**

| Botanical name | Herbal name | Dosage(g) | Voucher No. |
| --- | --- | --- | --- |
| [*Angelica* *gigas* Nakai](http://www.theplantlist.org/tpl1.1/record/kew-2639084) | *Angelica gigas* | 103.6 | 9-19-0332 |
| [*Paeonia* *lactiflora* Pall.](http://www.theplantlist.org/tpl1.1/record/kew-2560862) | *Paeonia lactiflora* | 103.6 | 9-19-0333 |
| *Cnidium* *officinale* Makino | *Cnidium officinale* | 103.6 | 9-19-0334 |
| [*Gardenia* *jasminoides* J.Ellis](http://www.theplantlist.org/tpl1.1/record/kew-88270) | *Gardenia jasminoides* | 103.6 | 9-19-0335 |
| [*Forsythia* *viridissima* Lindl.](http://www.theplantlist.org/tpl1.1/record/kew-369455) | *Forsythia viridissima* | 103.6 | 9-19-0336 |
| [*Mentha* *arvensis* L.](http://www.theplantlist.org/tpl1.1/record/kew-124385) | *Mentha arvensis* | 103.6 | 9-19-0337 |
| [*Zingiber* *officinale* Roscoe](http://www.theplantlist.org/tpl1.1/record/kew-273361) | *Zingiber officinale* | 103.6 | 9-19-0338 |
| [*Schizonepeta* *tenuifolia* (Benth.) Briq.](http://www.theplantlist.org/tpl1.1/record/kew-188177) | *Schizonepeta tenuifolia* | 103.6 | 9-19-0339 |
| *Saposhnikovia* *divaricata* (Turcz.) Schischk. | *Saposhnikovia divaricata* | 103.6 | 9-19-0340 |
| *Ephedra* *sinica* Stapf | *Ephedra sinica* | 103.6 | 9-19-0341 |
| [*Rheum* *undulatum* L.](http://www.theplantlist.org/tpl1.1/record/tro-26001337) | *Rheum undulatum* | 129.5 | 9-19-0342 |
| *Natrii sulfas* | *Natrii sulfas* | 129.5 | 9-19-0343 |
| [*Atractylodes* *japonica* Koidz. ex Kitam.](http://www.theplantlist.org/tpl1.1/record/gcc-114498) | *Atractylodes japonica* | 173.5 | 9-19-0344 |
| [*Platycodon* *grandiflorus* (Jacq.) A.DC.](http://www.theplantlist.org/tpl1.1/record/kew-354641) | *Platycodon grandiflorum* | 173.5 | 9-19-0345 |
| [*Scutellaria* *baicalensis* Georgi](http://www.theplantlist.org/tpl1.1/record/kew-188938) | *Scutellaria baicalensis* | 173.5 | 9-19-0346 |
| [*Glycyrrhiza* *uralensis* Fisch.](http://www.theplantlist.org/tpl1.1/record/ild-32406) | *Glycyrrhiza uralensis* | 173.5 | 9-19-0347 |
| *Talcum* | *Talcum* | 173.5 | 9-19-0348 |
| *Gypsum* | *Gypsum* | 259 | 9-19-0349 |

## 2.2 Supplementary Figures


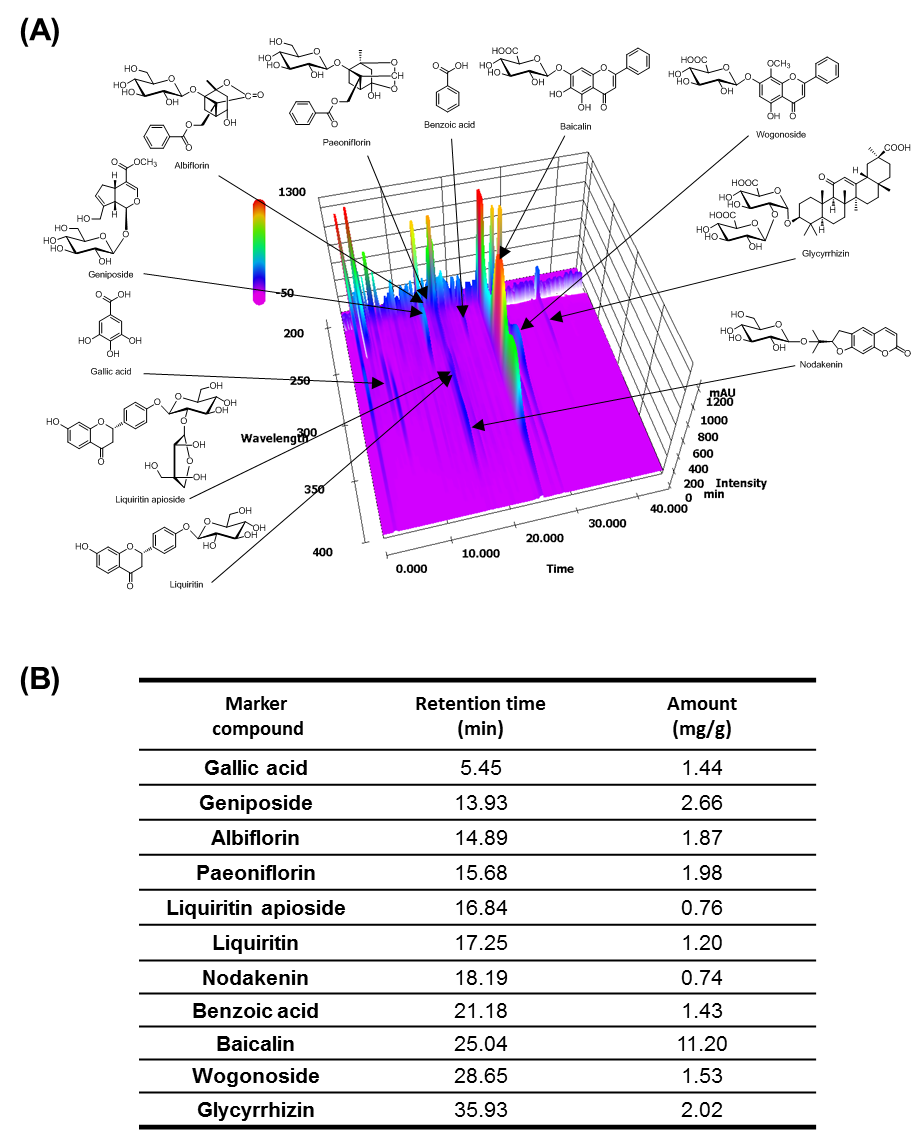


**Supplementary Figure 1.** **Three-dimensional chromatogram of BTS sample according to HPLC-PDA.** (A) Three-dimensional HPLC chromatogram of BTS. (B) Identification of marker components from BTS.


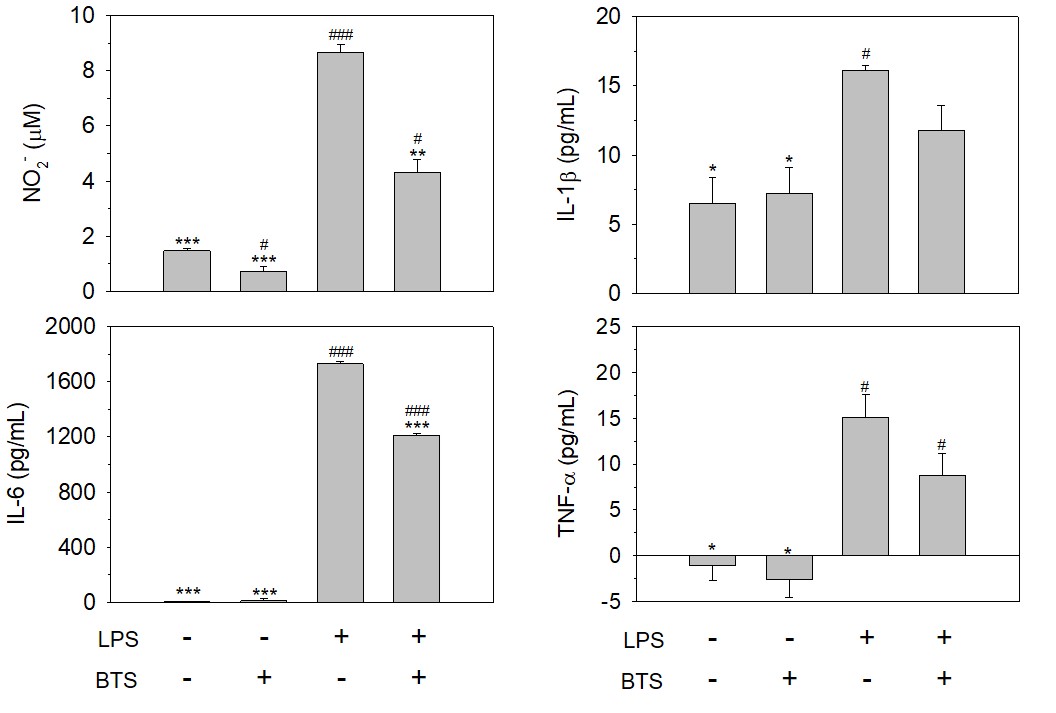


**Supplementary Figure 2.** Effect of BTS on the production of inflammatory modulators in mouse primary microglial (MMcg) cells. Primary MMcg cells were pretreated with BTS (final 400 μg/mL) for 1 h and then exposed to LPS (100 ng/mL). Twenty four hours after LPS treatment, the extracellular levels of NO, IL-1β, TNF-α, and IL-6 were determined. The data represent the mean ± SD of experiments in duplicate. The data represent the mean ± SD of experiments in duplicate (*t*-test: #p, ###p < 0.001 < 0.05 vs. untreated control; *p < 0.05, **p < 0.01, ***p < 0.001 vs. LPS-treated control).

**
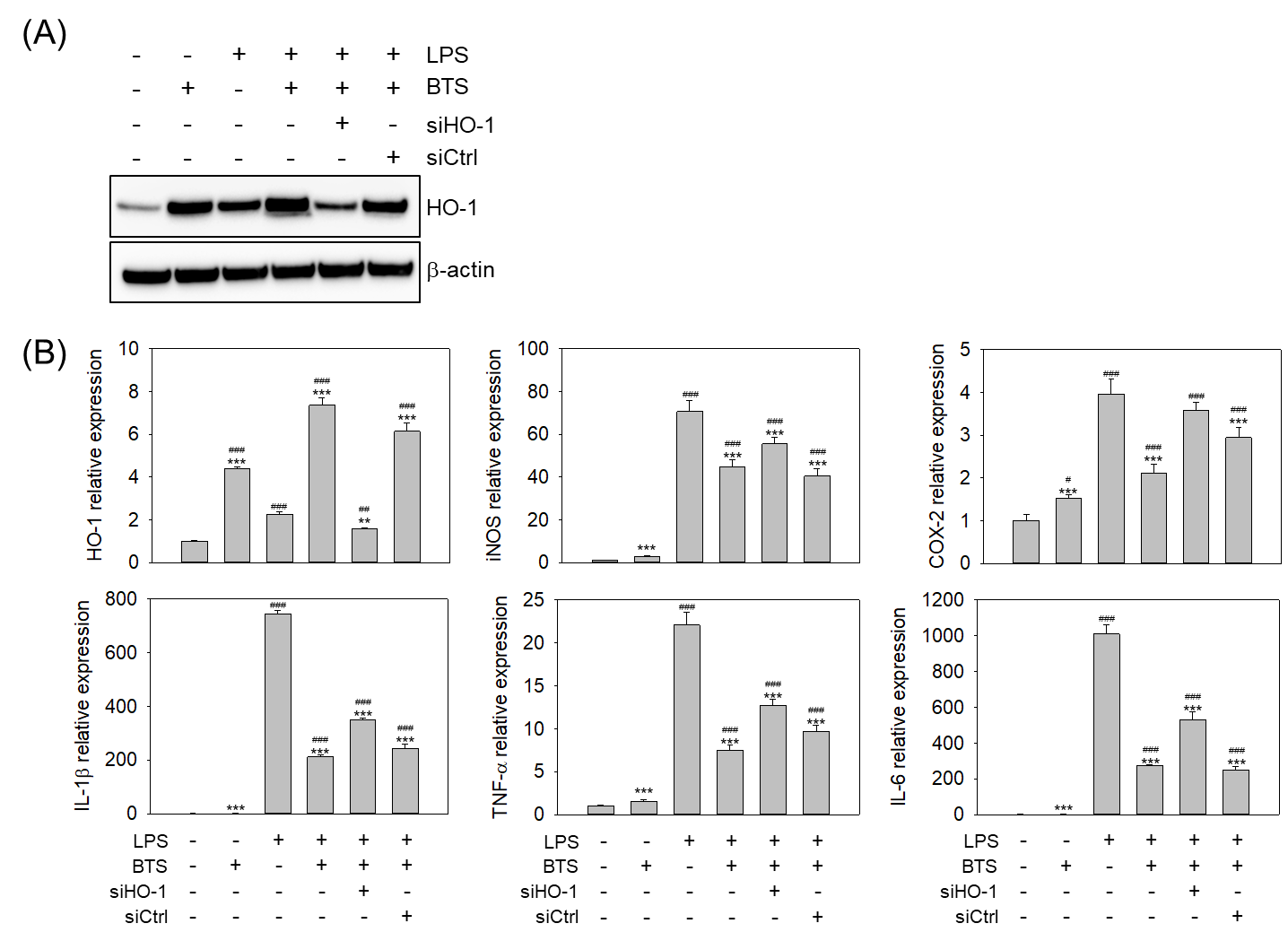
**

**Supplementary Figure 3.** HO-1 is a key regulator of BTS anti-inflammatory potential in BV2 cells. BV2 cells were transfected with siRNA targeting endogenous HO-1 (siHO-1) or control siRNA (siCtrl) at 10 μM. After 48 h, cells were pretreated with BTS (400 μg/mL) for 1 h and then exposed to LPS (100 ng/mL). (A) Intracellular HO-1 expression was determined by western blotting 24 h after LPS challenge. (B) The relative expression of *Hmox1*, *Nos2*, *Cox2*, *Il1b*, *Tnfa*, and *Il6* mRNAs was determined by quantitative real-time PCR using gene-specific primer sets as described in the Methods section. The data represent the mean ± SD of four replicates (one-way ANOVA: #p < 0.05, ##p < 0.01, ###p < 0.001 vs. untreated control; *p < 0.05, **p < 0.01, ***p < 0.001 vs. LPS-treated control).
